# Diseases at the back of the eye

**Published:** 2014

**Table:** 

Age-related macular degeneration (AMD)	Open-angle glaucoma	Diabetic retinopathy (DR)
History **Exudative AMD.** Distortion, rapid loss of central and reading vision **Atrophic AMD.** Gradual loss of central vision	Initially no symptoms, then gradual loss of the peripheral field of vision which can lead to loss of central vision	Initially no symptoms, then: **Maculopathy.** Gradual loss of central vision **Proliferative DR.** Sudden or gradual loss of vision
Examination **Exudative AMD.** Blood, or exudate, or scarring at macula **Atrophic AMD.** Atrophy of choroid and retinal pigment at macula	Pale and cupped disc, constricted visual fields, may have elevated intraocular pressure (IOP)	**Maculopathy.** Exudates near macula **Proliferative DR.** New vessels or vitreous haemorrhage
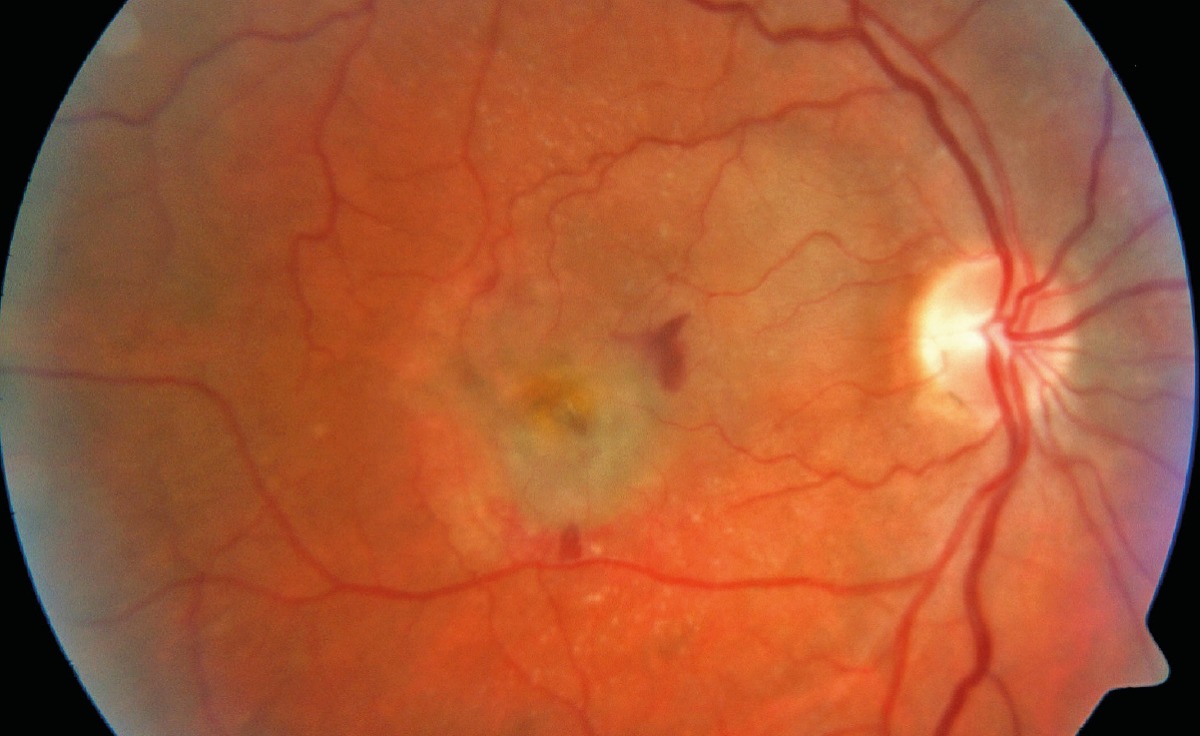 Exudative AMD	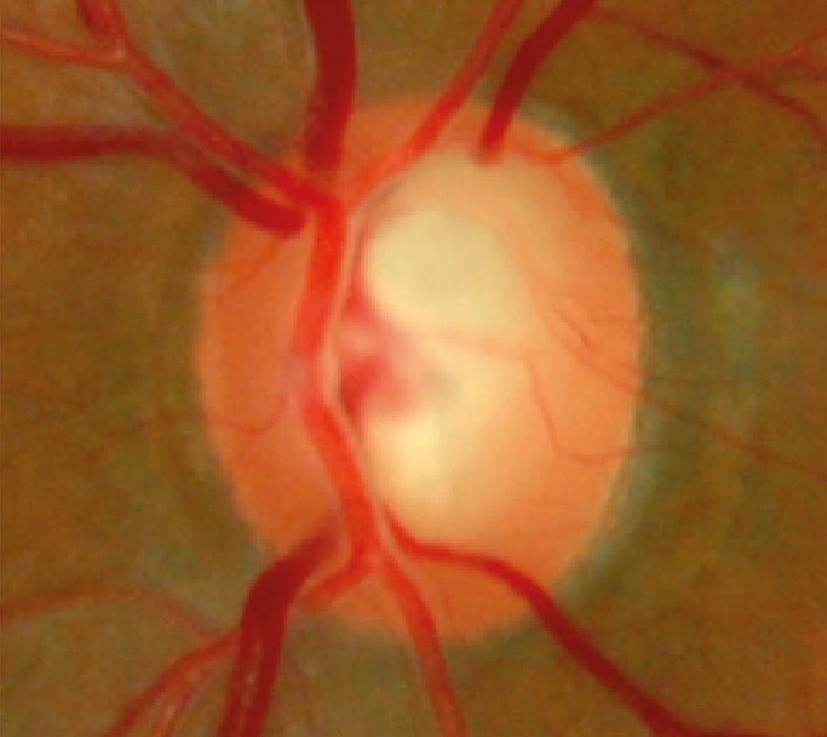 Early cupping	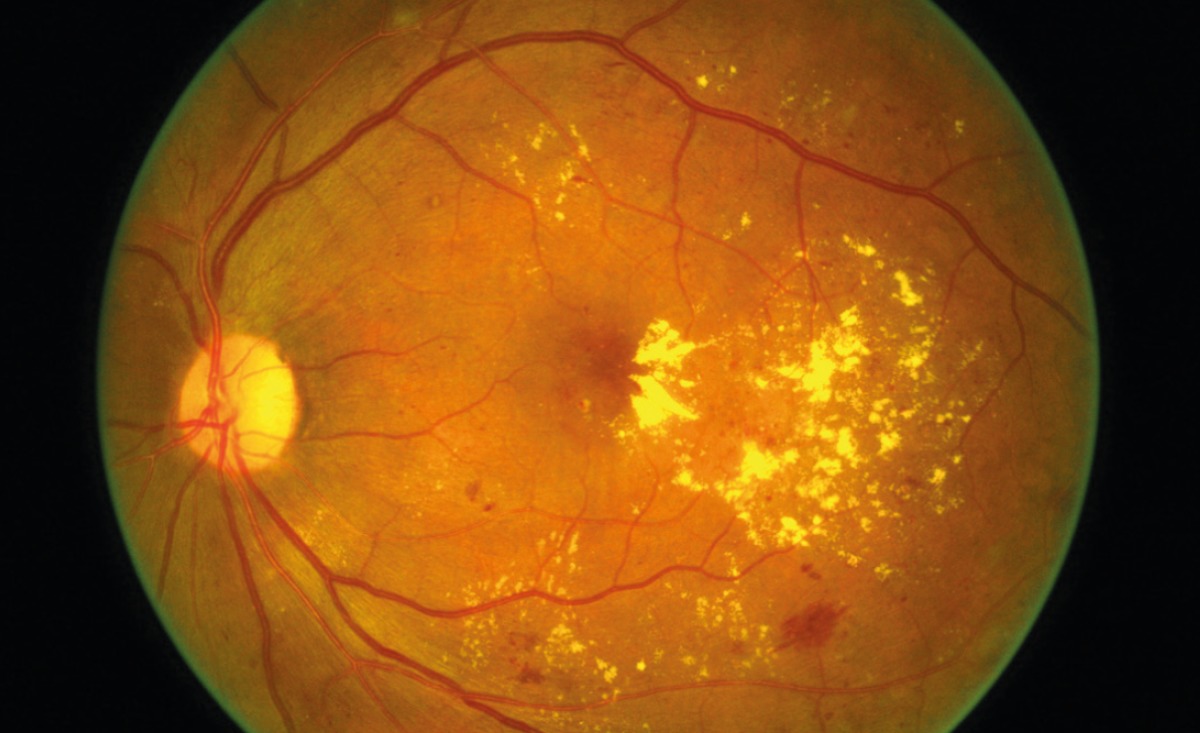 Diabetic maculopathy
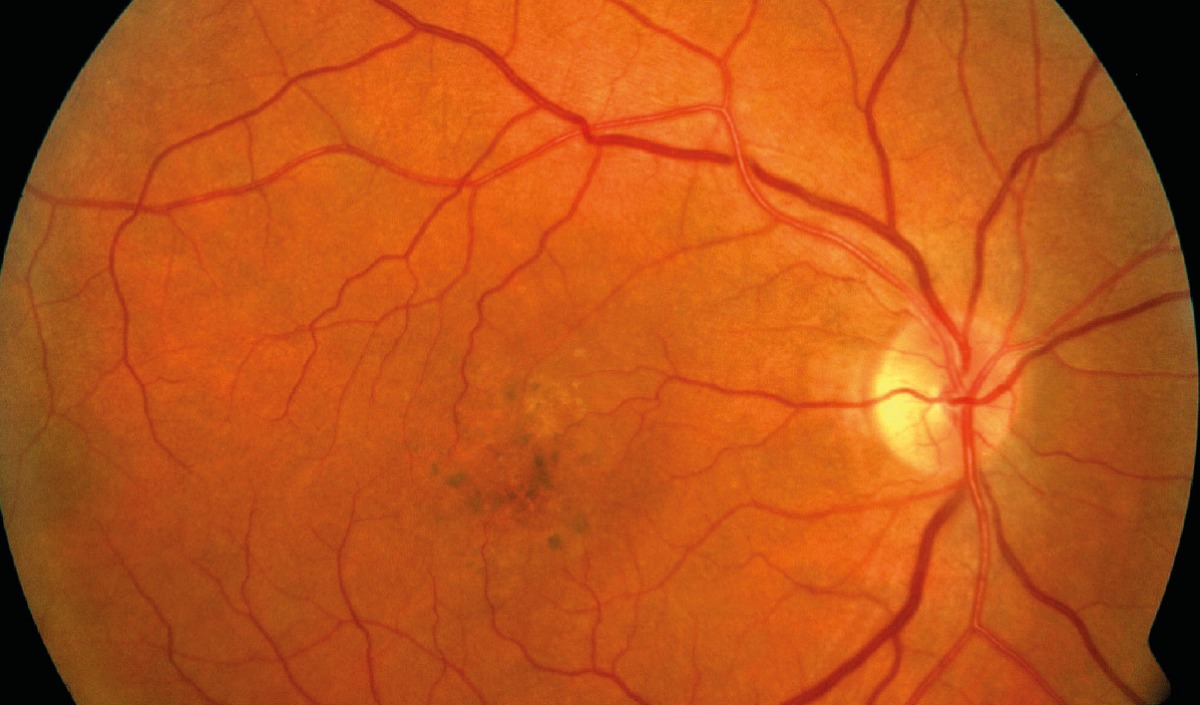 Early atrophic AMD	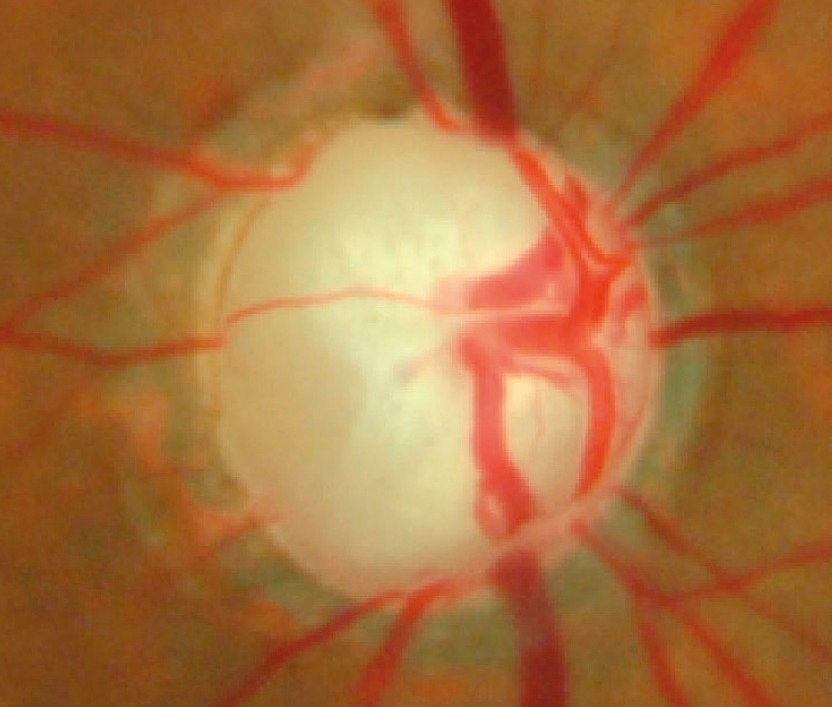 Cupped disc	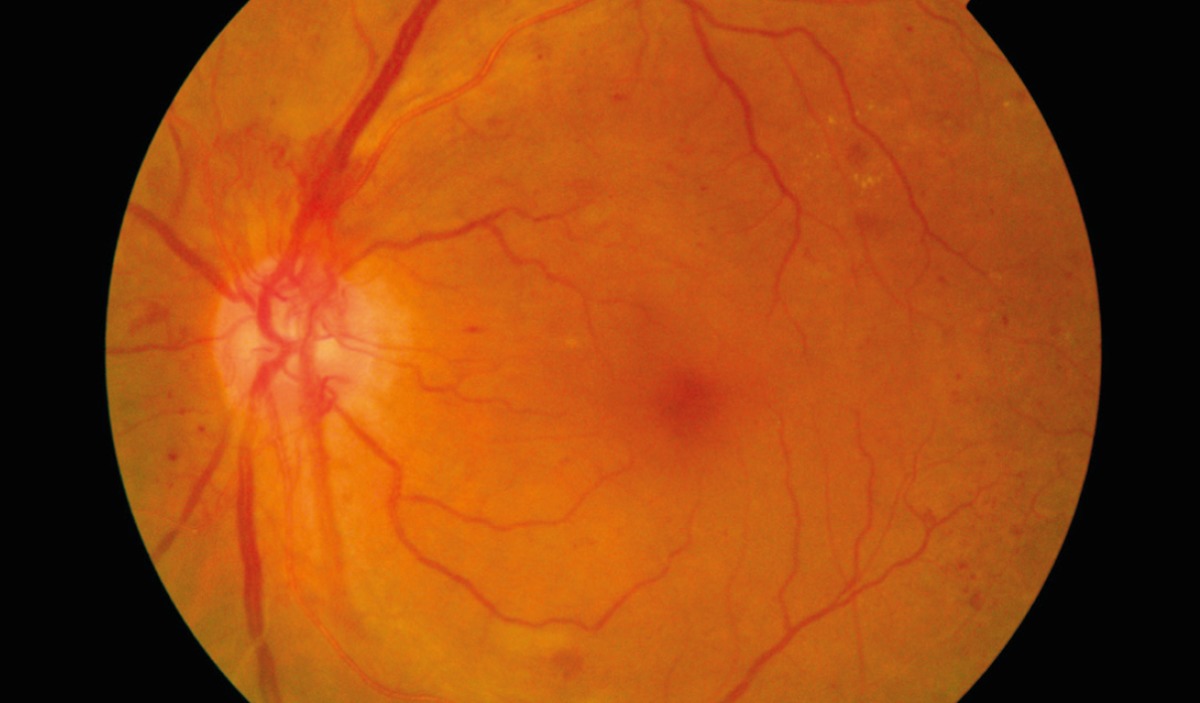 Diabetic new vessels (proliferative DR)
Management **Exudative AMD.** Refer for intravitreal injections (if available) if: Symptoms are present for less than three months Vision is better than counting fingers (CF) **Atrophic AMD.** No treatment is available, but patients may benefit from low vision aids	Treatment cannot improve sight, so refer only if the patient still has useful vision Aim to reduce IOP using: Daily eye drops Surgery Laser	**Maculopathy.** Refer for laser or intravitreal injections if vision is 6/60 or better **Proliferative DR.** Refer for laser if any new vessels or vitreous haemorrhage. May need vitrectomy if there is vitreous haemorrhage and/or poor vision
Information for Patients All three conditions are chronic, and cannot be completely cured. We expect anti-VEGF injections to improve vision in exudative AMD and diabetic maculopathy in most – but not all – patients. In glaucoma and proliferative DR, treatment will only prevent the condition getting worse. In order to manage these chronic and incurable disorders effectively, patients must attend the clinic regularly for the rest of their lives		
**Exudative AMD.** If suitable for treatment, patients will require three injections over three months. They are likely to need more injections after the initial three. Even if no further treatment is needed, they will need to attend the clinic every two months	The sight will not be improved by treatment, which aims to prevent further loss of vision. Eye drops must be used every day, and continued indefinitely. Surgery or laser may lower the IOP permanently but will require frequent examinations for the first three months	**Maculopathy.** The injection treatment is the same as for exudative AMD. Laser may lead to a more permanent cure, but still requires examination every 3-4 months	**Proliferative DR.** After laser, examine the patient every three months for the first year

